# Supplementary material for: A single case neuroimaging study of tickertape synesthesia
Source: Sci Rep. 2023 Jul 27;13:12185. doi: 10.1038/s41598-023-39276-2 (PMC10374523; doi:10.1038/s41598-023-39276-2)
Supplement: Supplementary file 1 — Supplementary Information. [file 41598_2023_39276_MOESM1_ESM.docx]

# Supplementary information

- Supplementary Figure S1
- Supplementary Figure S2
- Supplementary Figure S3
- Supplementary Figure S4
- Supplementary Figure S5
- Supplementary Figure S6
- Supplementary Table S1
- Supplementary Table S2

**
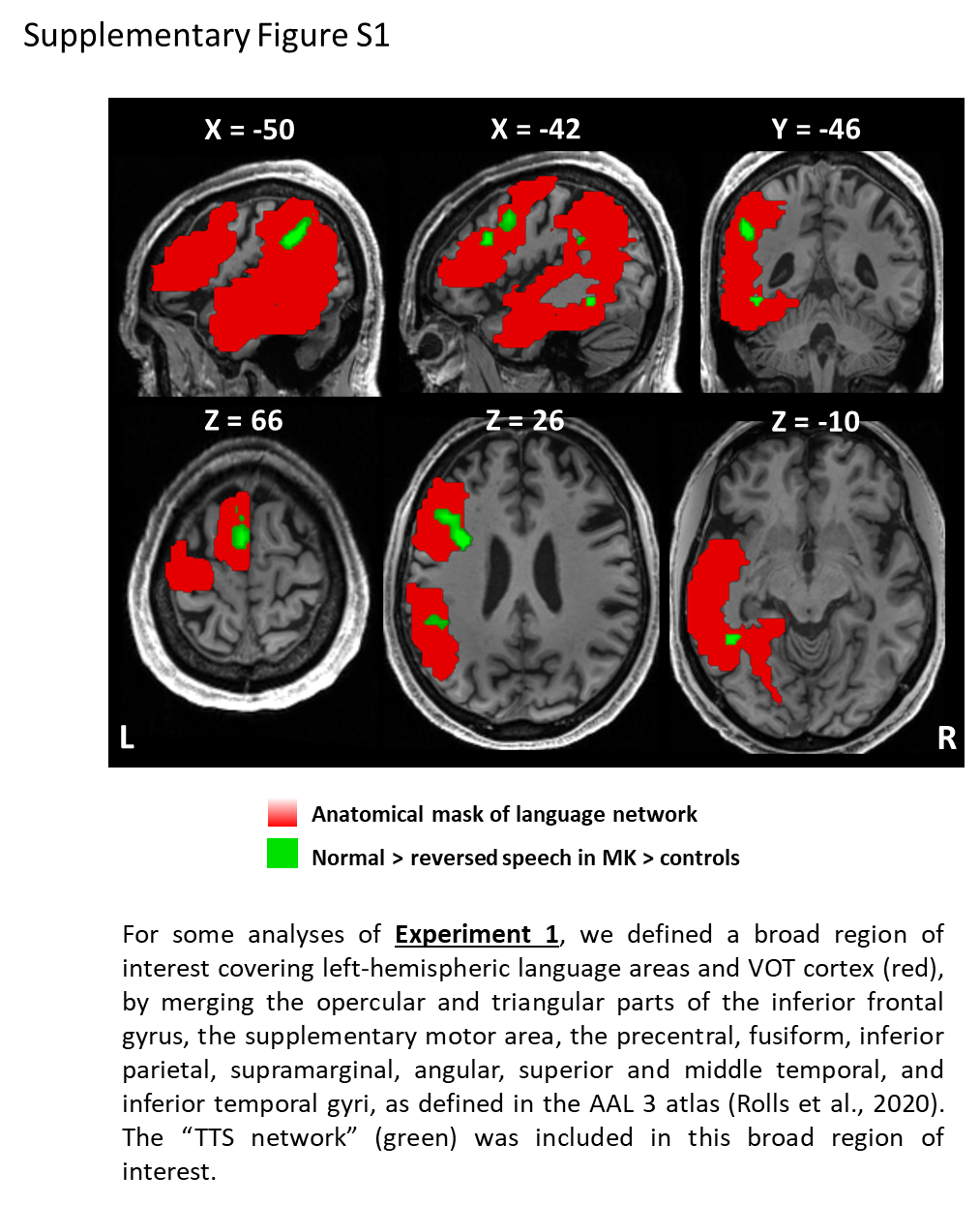
**

**
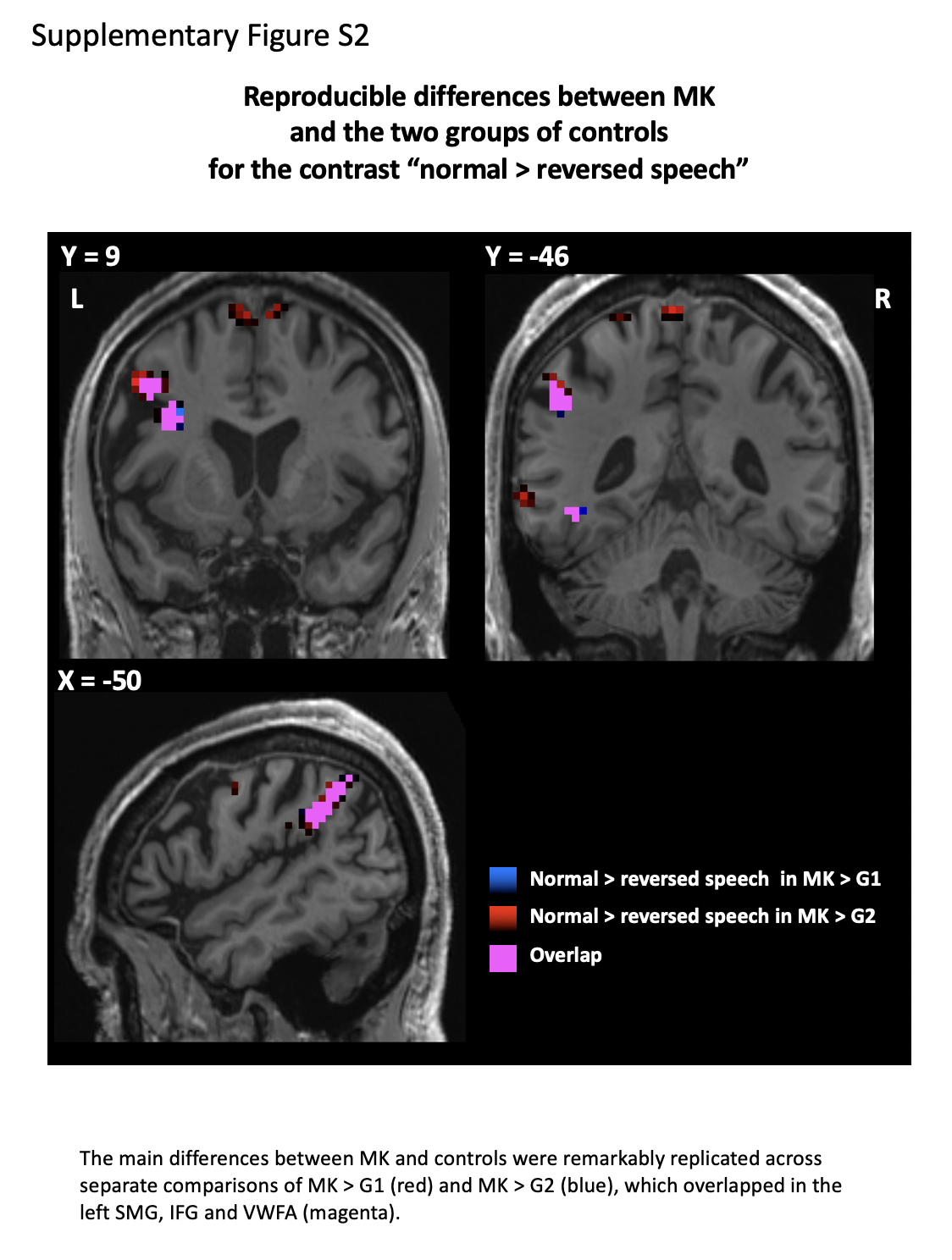

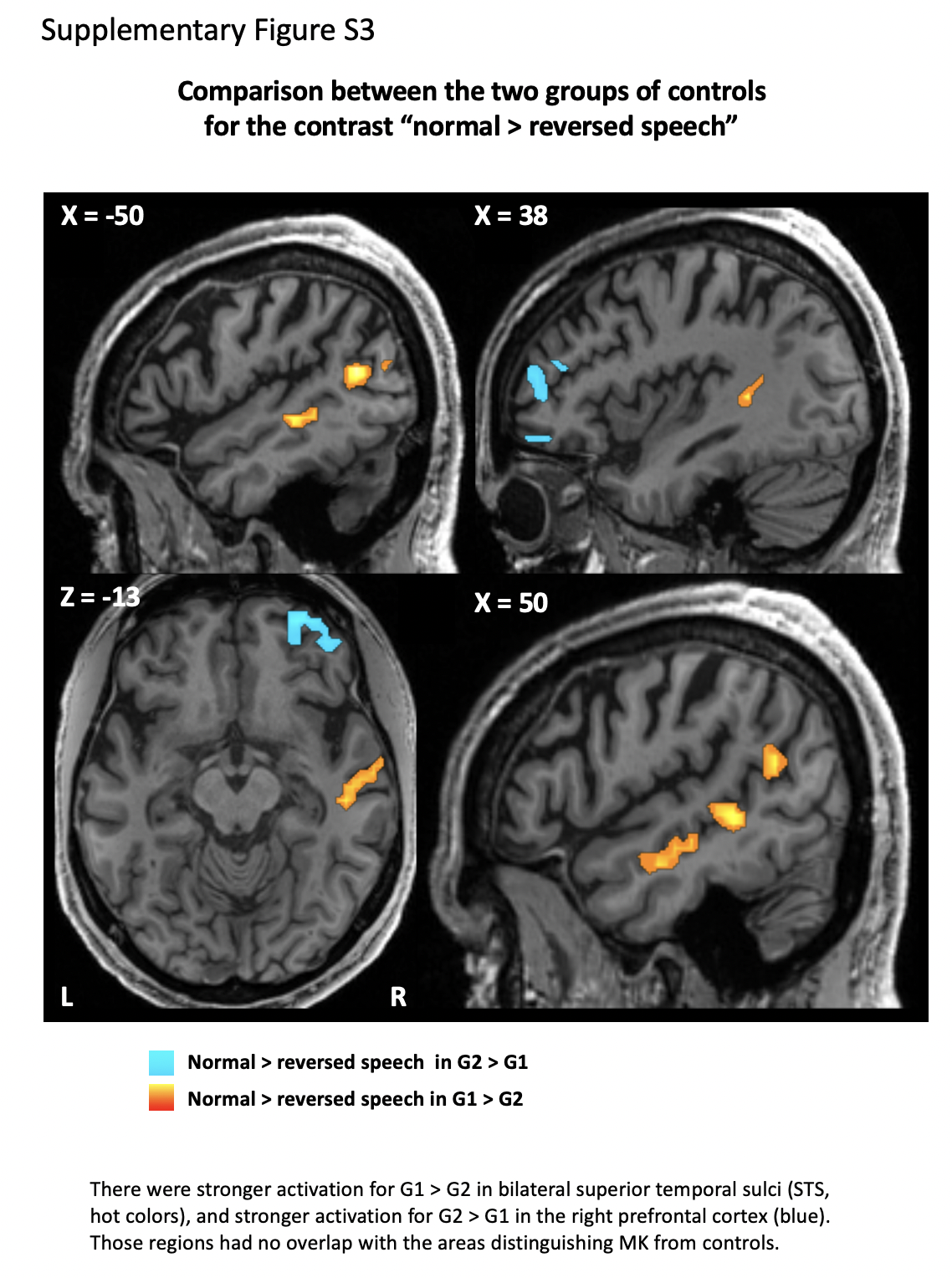
**

**
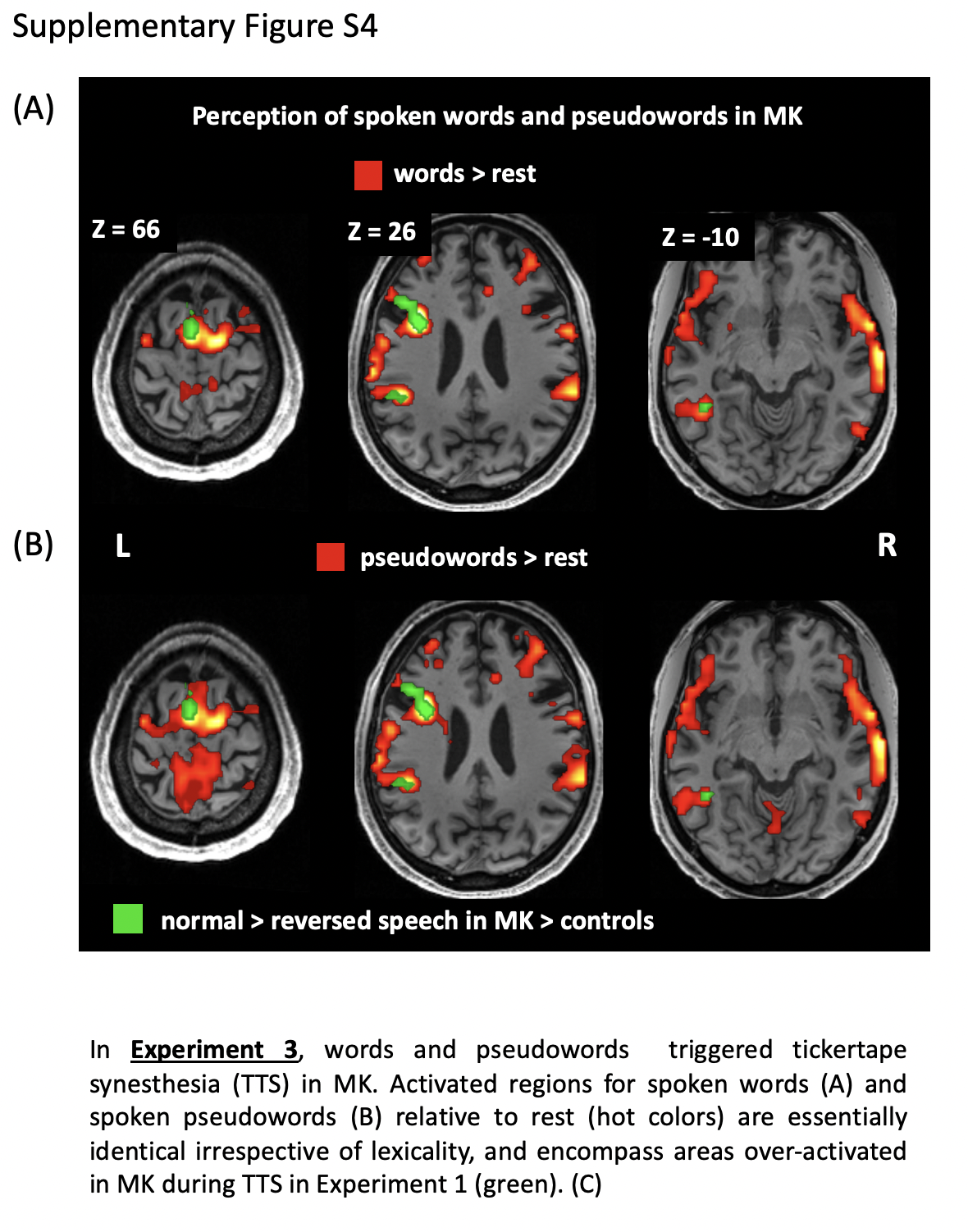

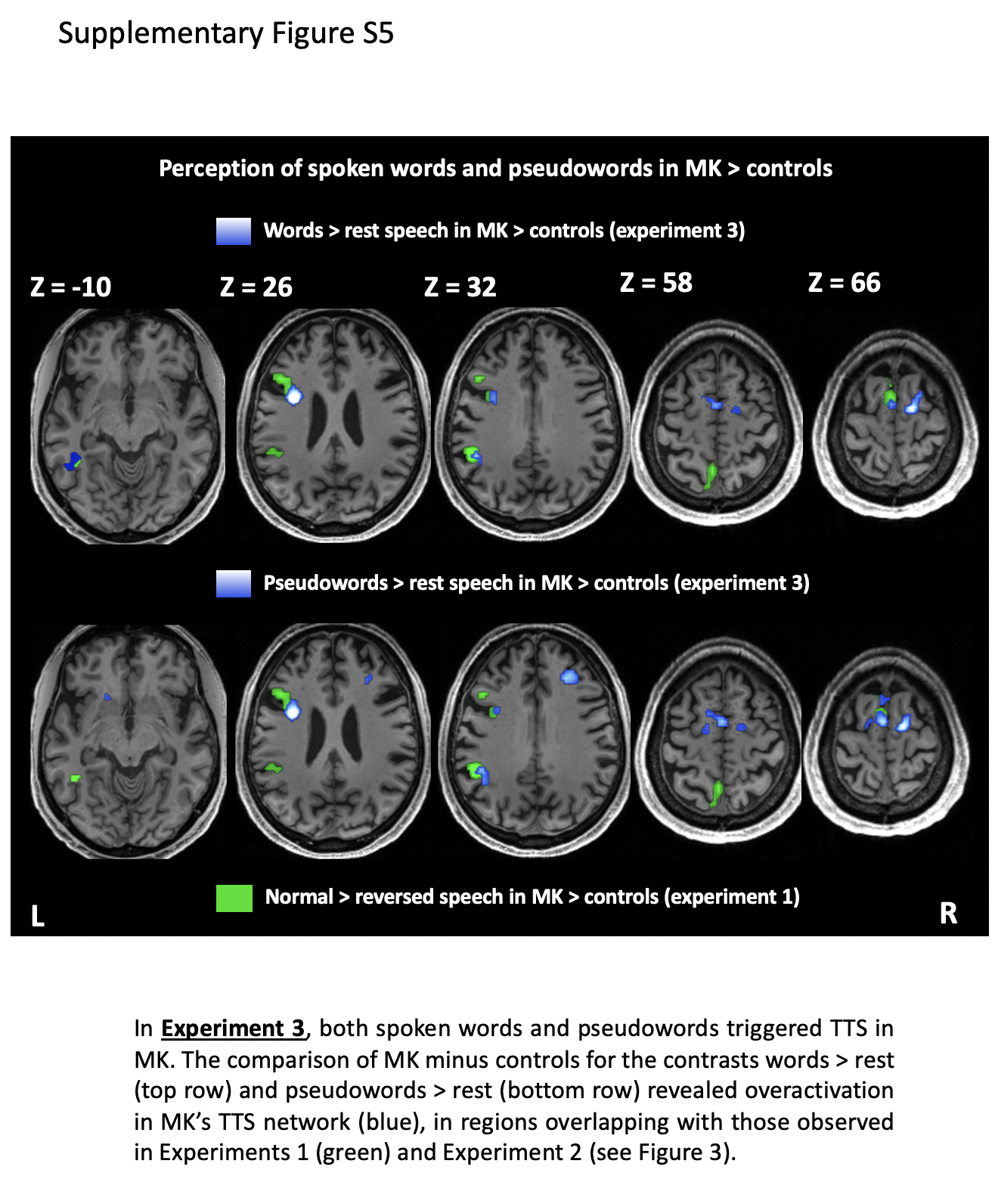

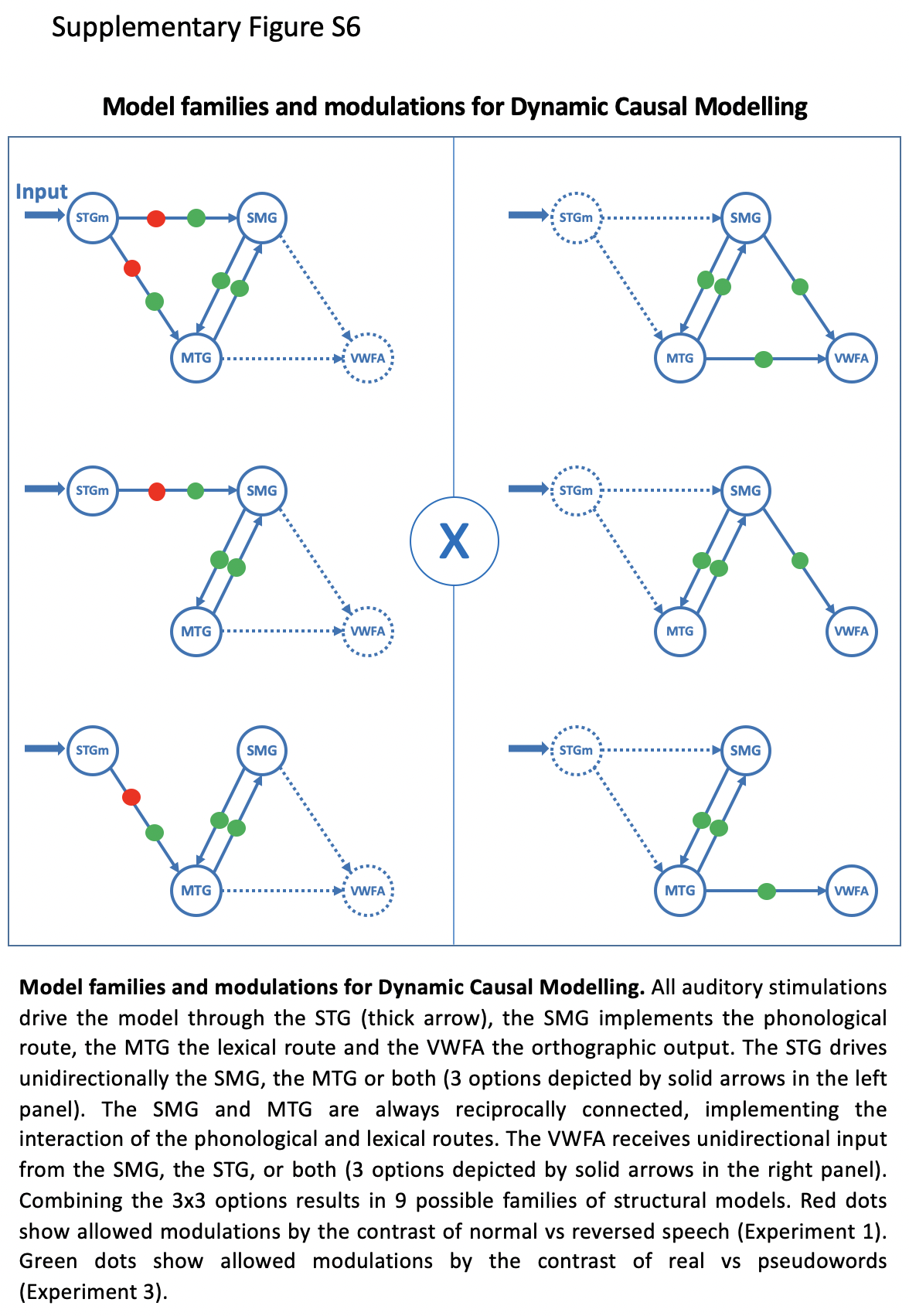
**

**Supplementary Table S1.**

**Experiment 1: Regions activated in controls when listening to reversed speech, and to normal > reversed speech.**

| Reversed speech > rest | | | | Normal > reversed speech | | | |
| --- | --- | --- | --- | --- | --- | --- | --- |
| Region | **Peak coordinates (MNI)** | **Z-score** | **Cluster-size** | **Region** | **Peak coordinates (MNI)** | **Z-score** | **Cluster-size** |
| Left STG | -48 -16 5 | >8 | 1263 | Left STS | -54 5 -16 | 7.56 | 4195 |
| Right STG | 51 -16 8 | >8 | 1318 | Right STS | 48 14 -22 | 6.40 |  |
| Right mesencephalic pedonculus | 18 -25 -4 | 5.21 | 42 | Left SMA | -39 11 53 | 5.32 | 96 |
| Right postero-inferior frontal gyrus | 42 11 20 | 5.16 | 211 | Right inferior frontal | 54 26 5 | 4.76 | 50 |
| Right SMA | 51 2 50 | 4.71 | 51 | Bilateral pre-cuneus | -6 52 38 | 4.73 | 177 |
| Left postero-inferior frontal gyrus | -33 14 29 | 4.04 | 48 | Left medial SMA | -6 5 65 | 4.52 | 50 |
|  |  |  |  | Bilateral orbitofrontal cortices | 0 59 -16 | 4.35 | 38 |

MNI, Montreal Neurological Institute; STG, Superior temporal gyrus; SMA, Supplementary motor area; STS, Superior temporal sulcus.

**Supplementary Table S2.**

**Experiment 2: Regions showing preferential activations for categories of visual stimuli.**

| Contrast | Region | Peak coordinates (MNI) | Z_max_ |
| --- | --- | --- | --- |
| Words > Houses and Faces | Left STG | -63 -22 5 | > 8 |
|  | Left fusiform gyrus | -45 -52 -10 | > 8 |
|  | Right STG | 69 -31 11 | 7.71 |
|  | Left inferior frontal gyrus | -33 8 23 | 6.85 |
|  | Left supramarginal gyrus | -48 -40 32 | 6.29 |
|  | Left precuneus | -9 -73 59 | 4.72 |
|  | Right cuneus | 12 -97 14 | 4.6 |
|  | Left SMA | -6 8 62 | 4.43 |
| Faces > Houses and Words | Right peristriate area | 51 -70 -1 | 6.38 |
|  | Right fusiform gyrus | 42 -55 -13 | 5.44 |
| Houses > Faces and Words | Right visual association area | 30 -88 8 | > 8 |
|  | Left visual association area | -33 -85 23 | > 8 |
|  | Left parahippocampal gyrus | -30 -37 -16 | > 8 |
|  | Right parahippocampal gyrus | 30 -49 -10 | 6.09 |
| Tools > (Words, Houses and Faces) | Left lateral occipital cortex | -42 -67 2 | > 8 |
|  | Right lateral occipital cortex | 45 -64 -4 | > 8 |
|  | Left fusiform gyrus | -30 -49 -13 | 5.08 |
| Bodyparts > (Words, Houses and Faces) | Right lateral occipital cortex | 45 -64 -4 | > 8 |
|  | Left lateral occipital cortex | -42 -67 2 | > 8 |
|  | Left fusiform gyrus | -30 -52 -13 | 5.1 |

MNI, Montreal Neurological Institute; STG, superior temporal gyrus; SMA, supplementary motor area.
